# Design and Application of A Bioluminescent Biosensor for Detection Of Toxicity Using Huh7-CMV-Luc Cell Line

**DOI:** 10.22037/ijpr.2019.1100687

**Published:** 2019

**Authors:** Nazanin Rahimirad, Saeedeh Kavoosi, Hadi Shirzad, Majid Sadeghizadeh

**Affiliations:** a *Department of Genetics, Faculty of Biological Sciences, Tarbiat Modares University, Tehran, Iran.*; b *Department of Genetics, Faculty of Medical Sciences, Tarbiat Modares University, Tehran, Iran.*

**Keywords:** Toxicity, Whole cell biosensor, Huh7 cell line, CMV enhancer, Luciferase assay

## Abstract

Cell-based biosensors (CBBs) are becoming important tools for biosecurity a lications and rapid diagnostics in food microbiology for their unique capability of detecting hazardous materials. Pollutants, such as heavy metals and chemicals, are now considered as a global threat and are associated with detrimental health outcomes. Fast and accurate detection of pollutants is essential to reduce these threats. In this study, the enhancer sequence of human cytomegalovirus (hCMV) IE genes cloned upstream of *luc* gene in PGL4.26 plasmid, in order to increase basal luciferase activity. This recombinant vector was transfected into Huh7 cell line and after 21 days of treatment with Hygromycin B selectable marker, stable cell line was generated. After several passages, cells containing this vector showed high luciferase activity in normal conditions without any induction following to overexpression of *luc* gene. Huh7-CMV-luc cell line was able to detect the slightest changes in ATP level, due to the effect of different toxins on the cell which disrupt cellular respiration and ATP production processes. In order to investigate the sensitivity of the cell line, the cells were incubated with 0.1-10 μM of chemical toxins affecting ATP production. These toxins affect luciferase activity in a dose dependent manner, with maximal sensitivity approximately about 0.2 μM to toxin concentrations. Additionally, this biosensor provided a rapid detection as early as 4 h in response to the toxicants. Whole cell biosensors like huh7-CMV-luc cell line can be considered as a powerful tool for the sensitive and efficient monitoring of general toxins, drugs, and environmental pollutants.

## Introduction

The dynamic worldwide agri-food market has produced increased chemical, biological, and physical threats to food products, which endangers food safety and consequently general health (1). According to the World Health Organization (WHO), each year millions of people around the world are affected by foodborne diseases. Biological risks produced by bacteria, viruses and bio toxins, chemical substances (e.g., additives), food nutrients used to add product value, pesticides, veterinary drugs residues and processing operations may constitute risks to consumers’ health (2). Since the concern over the pollution risk to food and drinking water from industry and agriculture is growing, the need for a powerful functional tool to allow for the rapid, effective, and efficient detection of hazards and threats inherent to safety and quality of food have emerged.

Biosensors, which are an important option in the food sectors to control production processes and ensure food quality and safety by reliable and cost effective procedures, have gained enormous attention and developed in different a lications, including bioprocess control, food quality control, agriculture, military, defense against bioterrorism and in particular, for medical a lications in recent years. They can be considered as a subclass of chemical sensors in which a biological mechanism is used for analyte detection (3). Biosensors are self-contained integrated devices, consisting of a biological recognition element in direct contact with a transduction element, which converts the biological recognition event into a useable output signal. Biosensors can be classified to different groups according to their bioreceptor element involved in the biological recognition process such as enzymes, immunoaffinity recognition elements, whole cells of micro-organisms, plants or animals, or DNA fragments (4). The most important advantage of whole cell biosensors in comparison with other subgroups is that a live, intact cell is used as the biological entity. In this powerful new a roach we are able to detect very complex series of reactions that can exist only in an intact, functioning cell by very simple means and measure bioavailability of toxins and biological effects rather than total concentrations obtained by traditional analytical techniques (5).

The aim of this study was to establish a sensitive, stable reporter cell line using a “lights off” strategy which is based on the measurement of a decrease in light emission following exposure to the sample. According to this strategy, we had to use luciferase gene under the transcriptional control of a strong constitutive promoter in order to increase the expression of *luc* gene and luciferase activity. For this purpose, the enhancer sequence of human cytomegalovirus (hCMV) IE genes was cloned upstream of *luc* gene. Immortalized cells derived from human hepatocarcinoma cell line (Huh-7) was transfected by this recombinant construct. Stable cell line achieved using the a ropriate dose of hygromycin B antibiotic after 21 days. The reporter cell line was challenged with a range of compounds, to evaluate the decrement in luciferase activity (which ha ens as a consequence of ATP reduction followed by exposure to toxins). The reporter cell line is sensitive enough to screen chemicals with toxicity risk. 

## Experimental

*Chemicals*

Fetal bovine serum (FBS) was purchased from Invitrogen/Life Technologies (Carlsbad, CA, USA). Dimethyl sulfoxide (DMSO) was the products of Sigma-Aldrich (St. Louis, USA). Hygromycin B was purchased from Sigma-Aldrich (St. Louis, USA). Lipofectamine 2000 reagent was purchased from Invitrogen/Life Technologies (CA, USA).

*Generation of recombinant vector*

Primers for the enhancer sequence of IE (immediate-early) genes of human cytomegalovirus (hCMV) were designed in order to proliferate a fragment of about 420 bp length including KpnI-HindIII recognition sites. The amplified fragments were purified on a 1.5% agarose gel and cloned at the KpnI-HindIII sites of pGL4.26 using standard protocols. The orientation and sequences of this element were confirmed by sequencing of the plasmids.

*Development of a toxicity sensitive luciferase reporter plasmid*

The toxicity reporter plasmids were generated using the pGL4.26-minimal promoter vector (Promega, UK, Southampton, United Kingdom) containing a minimal TATA promoter upstream of the firefly luciferase gene (*luc*). Enhancer element of CMV IE genes was inserted into the pGL4.26 [minP] multiple cloning site upstream of the *luc* gene. Consequently, TOP10 competent cells were transformed with the recombinant DNA for amplification. Engineered vector contains one copy of enhancer sequence of HCMV immediate-early genes that have been inserted through KpnI-HindIII restriction sites upstream of the promoter-luc + transcriptional unit. Five positive clones were sequenced using RV3 primer (*Rhinovirus* universal primer).

*Cell viability assay*

Cell viability was assessed by methyl thiazol tetrazolium (MTT) assay (Sigma-Aldrich, St Louis, USA) according to the manufacturer’s instruction. Briefly, the cells (1 × 10^4^) were cultured overnight in a 96-well plate. Afterwards, the medium of each well was replaced by 200 μL fresh medium plus 50 μL of the MTT solution (5 mg/mL in PBS). The plates were incubated at 37 °C for 4 h. The absorbance being proportional to cell was subsequently measured at 570 nm in each well using an enzyme linked immunosorbent assay plate reader (BioRad 680, USA). All experiments were performed in triplicate, and the relative cell viability (%) was calculated as a percentage relative to the untreated control cells.

*Generation of stable cell line*

The Huh7-CMV-luc containing the Hygromycin B selectable marker was stably transfected into the Huh7 cells using the Lipofectamine® 2000 reagent. According to the LD50 value, the transfected cells were selected using 450 μM Hygromycin B (Invitrogen/Life Technologies, CA, USA) in the media for 21 days. The Hygromycin B-resistant clones were isolated and screened by measuring their basal and inducible luciferase activity at different concentration of toxins. Positive clones showed high luciferase activity. The cells were maintained in growth medium containing 450 μM hygromycin for further analysis.

*Cell culture conditions*

The human hepatocarcinoma cell line (Huh-7) was purchased from National Cell Bank, Pasteur Institute of Iran and were grown in Dulbecco′s Modified Eagle′s Medium (DMEM) (Invitrogen/Life Technologies, Carlsbad, CA, USA). The cells were su lemented with 10 % fetal bovine serum, 100 U/mL penicillin, and 100 mg/mL streptomycin (Invitrogen/Life Technologies, Carlsbad, CA, USA). The cells were grown at 37 °C in a humidified atmosphere of 5% carbon dioxide. the cells were tripsinized/sub cultured every 2 to 3 days.

*Chemical exposure*

Sterile stock solutions of chemical food toxins were prepared in DMEM just before use. Briefly, the cells were seeded at a density of 2 × 10^5^ per well in 24-well microtiter plates, and incubated until the cells reached 70–80% confluence. Following overnight recovery, the culture medium was replaced by the fresh DMEM su lemented with antibiotics along with a range of chemical concentrations in triplicate for 4 h, 6 h, 12 h and 24 h to estimate the luciferase shortest induction time.

*Huh7-CMV-luc reporter-gene assay*

Huh7 cells were seeded in 24-well plates at a density of 2 × 10^5^ cells per well and grown overnight. Consequently, the cells were transiently transfected with the recombinant reporter plasmids. The plasmid pGL4.26 without the CMV enhancer fragment was considered to control the performance of the construction. Transfection was done by Lipofectamine 2000® (Invitrogen, Carlsbad, CA, USA) reagent in triplicate according to the manufacturer’s instructions. Following transfection the culture medium was replaced 24 h later with the fresh growth medium containing 0.1 to 1o μmol/L Nitrite and lead which was prepared immediately before each experiment. The cells were left for 24 h to respond to the toxins, and then the firefly luciferase activities in their lysates were monitored.

*Luciferase assay*

Luciferase assays were performed following the manufacturer’s instruction (Promega). The cells were washed twice with phosphate buffered saline (PBS). Each well received 50 μL lysis buffer (Promega, UK, Southampton, United Kingdom) after removing PBS. The cell lysates were harvested and spin for 5 min. The cell lysates (50 μL) were added to 10 μL luciferase assay reagent (Promega, UK, Southampton, United Kingdom). Luciferase bioluminescence measurements were performed at room temperature using a luminometer (Sirius tube Luminometer, Berthold Detection System, Germany). The activity was expressed as relative light units (RLU) emitted from total assays and it was calculated versus background activity.

**Figure 1:**
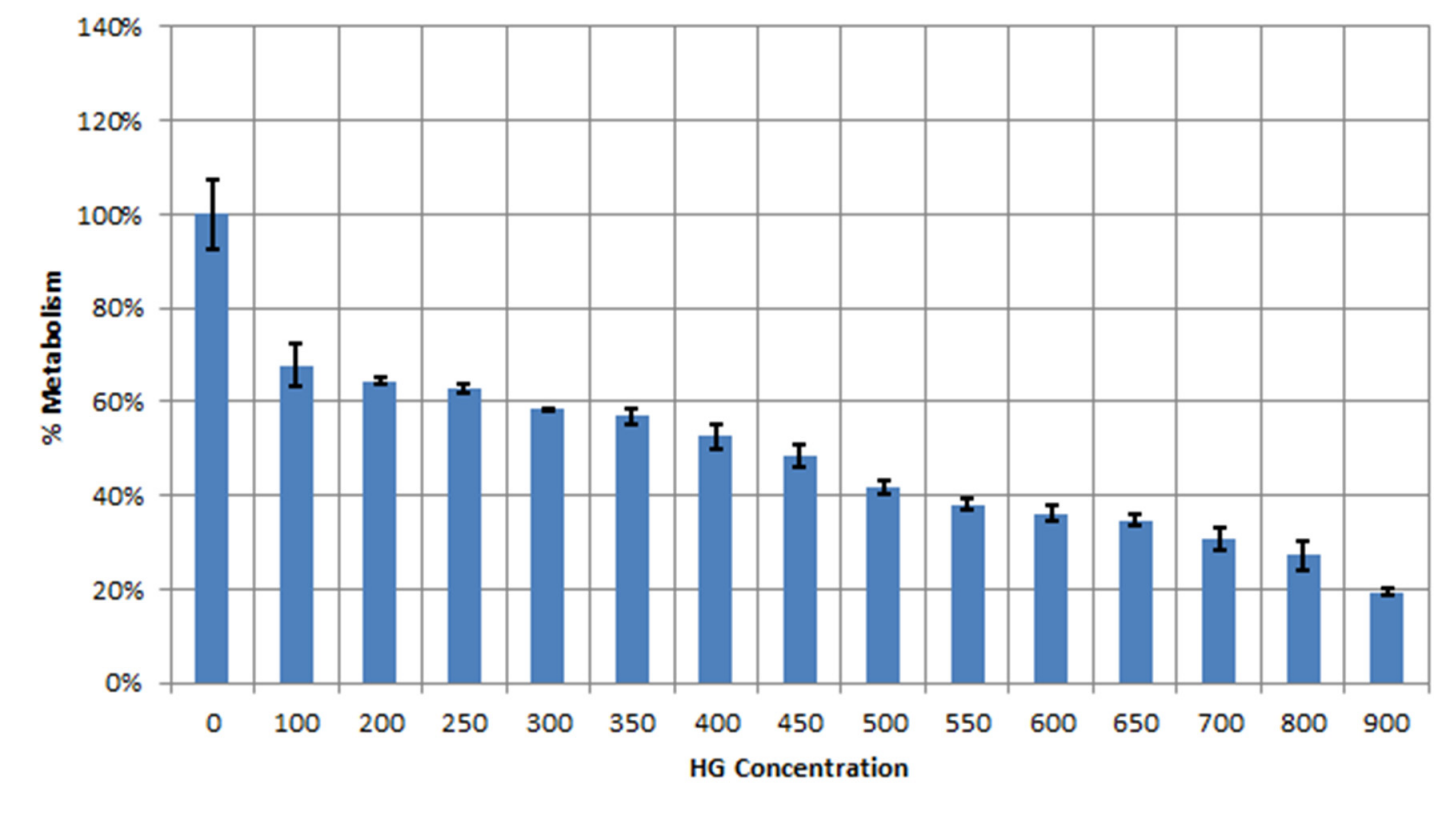
MTT assay indicating the inhibitory effect of Hygromycin B on human hepatoma cells (Huh7). Data showed LD50 value of450 μM Hygromycin B for Huh7 cell line

**Figure:**
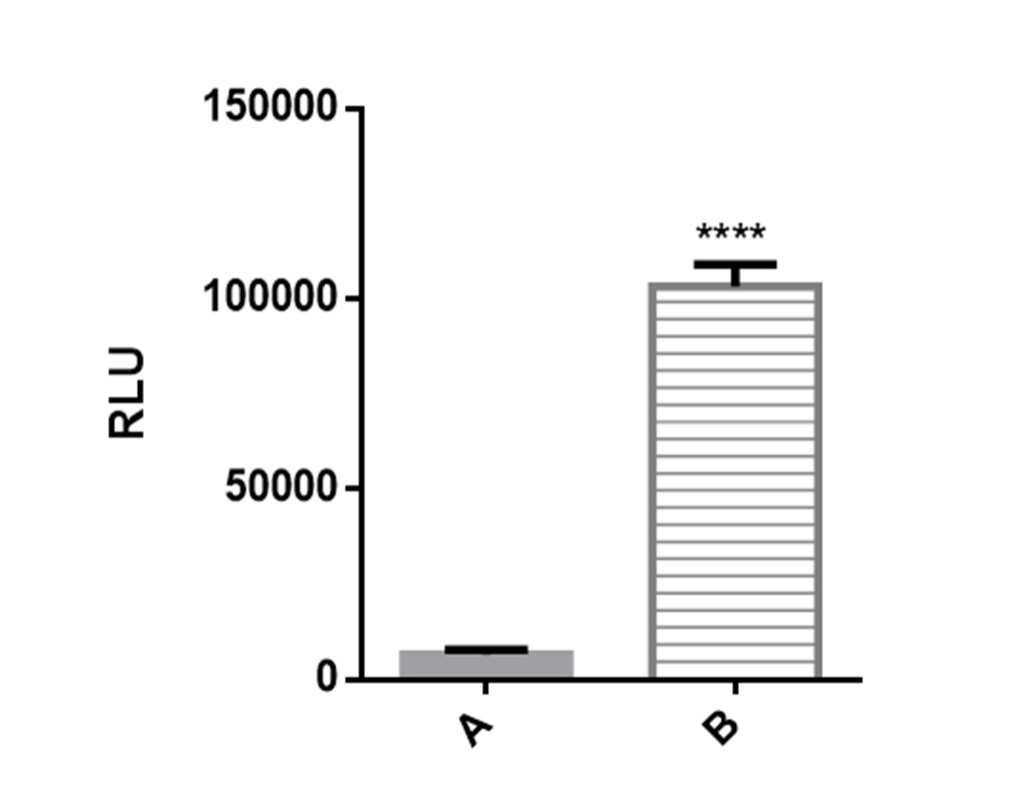
**Figure 2**. Luciferase reporter activity in transiently transfected Huh7-CMV-luc cells. Recombinant cells were seeded overnight in 24 well plates at 2 × 105 cells per well. After 24 h luciferase activity was assessed by measuring luciferase activity in cell lysates. Transfected cells with recombinant vector including CMV enhancer element (column B) showed significant increase in luciferase activity compared to control cells which have PGL4.26 plasmid with minimal promoter lacking CMV fragment (column A).

Notes: Data expressed as mean ± SD; ****, *p *< 0.0001 compared to control.

**Figure 3:**
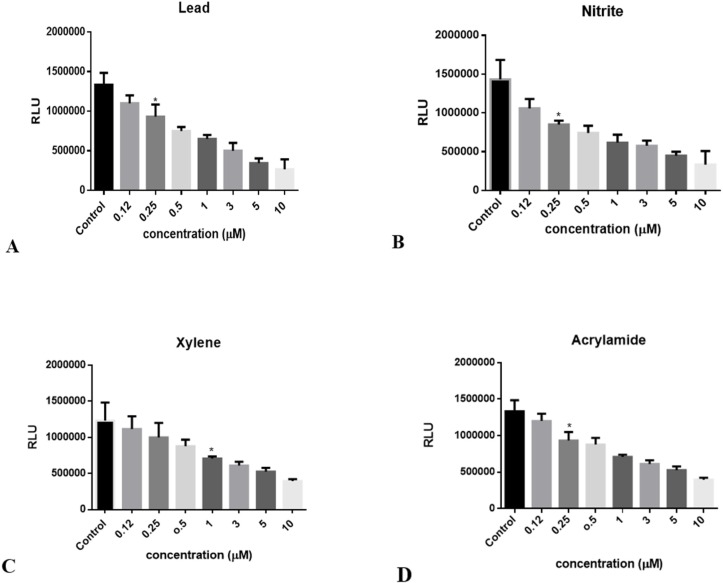
Decrement in luciferase activity following to ATP reduction in Huh7-CMV-luc stable cell line exposed to: **(A) **lead concentrations (0.1 to 10 μM), **(B) **Nitrite concentrations (0.1 to 10 μM), **(C) **Xylene concentrations (0.1 to 10 μM), and **(D) **Acrylamide concentrations (0.1 to 10 μM). Luciferase activity was measured after 24 hours. The numbers in the left column represent the relative luciferase activities. Luciferase activity was decreased following treatment with increasing concentrations of toxins. Each bar shows the mean ± SD; *, *p *= 0.0327 for (A), *p *= 0.0170 for (B), *p *= 0.0228 for (C), and *p *= 0.0224 for (D) compared to control

**Figure 4:**
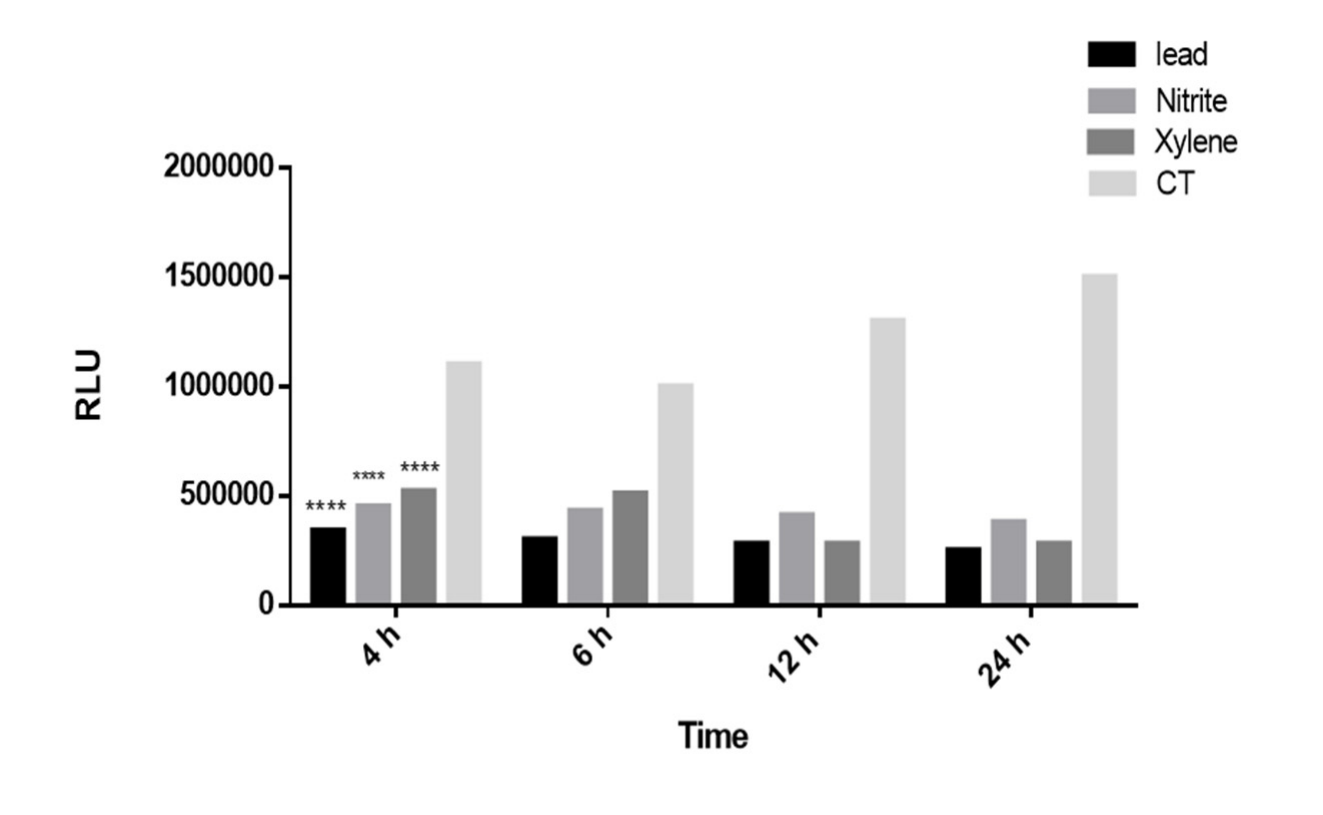
The time course of luciferase activity induction. The Recombinant cell line was incubated with Lead, Nitrite and Xylene (5 μM) at 37 °C for the indicated time after which luciferase activity in cell lysates was measured as described under the “Methods” section. Luciferase activity was expressed as RLUs in recombinant Huh7 cell line. The luciferase induced significantly after 4 h as the shortest induction time. Each bar shows the mean ± SD; ****,*P *< 0.0001 compared to control

*Statistical analysis*

All experiments were conducted in triplicate, and the results were expressed as mean ± SD (standard deviation). One-way analysis of variance (ANOVA) was used to assess the statistical analysis, and *p* < 0.05 was considered to be significant. The data were analyzed using Microsoft Excel software and GraphPad Prism software.

## Results

The "huh7-CMV-luc biosensor" was obtained by stably transfection of recombinant PGL4.26 plasmid which contains *luc* gene expressing luciferin substrate essential for luciferase activity to huh7 cell line. The cells were treated with LD_50_ dose of hygromycin B selectable marker for 21 days. After several passages of the cells, for detection of slightest toxicant agents in the environment, luciferase assay was performed in the presence of increasing dosage of different toxins (from 0.1 µM to 10 µM). Decrease in luciferase activity was reported according to toxicant exposure.

*Cytotoxic studies*

Data from MTT assay clearly showed LD_50_ value of 450 μM Hygromycin B antibiotic for huh7 cells. (Figure 1).

*Transient transfection and luciferase assay analysis*

Prior to stable transfection, high level of luciferase activity of the plasmid pGL4.26-CMV-luc was confirmed in transient transfection experiments. Transiently transfected cell line with PGL4-CMV-luc recombinant vector showed significantly high luciferase activity compared to the huh7 cells transfected with PGL4.26 plasmid. It was found that basal luciferase activity has significantly increased due to insertion of enhancer element of CMV promoter upstream of *luc* gene minimal promoter (Figure 2. Column B). 

The cells transfected with PGL4.26 (which contains only a minimal promoter upstream of *luc* gene and lacks enhancer element) had very low luciferase activity (Figure 2. Column A), whereas a significant induction of luciferase activity was observed in the cells transfected with pGl4.26-CMV-Luc as a result of enhancer element of a strong constitutive promoter which caused an a roximately 10,000-fold increase in luciferase activity compared to the control (*p* < 0.001).

*Generation of stable biosensor and detection of toxicity*

The pGL4.26-CMV-luc plasmid containing the Hygromycin B selectable marker was stably transfected into Huh7 cells. The cells were selected in media containing 450 μM Hygromycin B for 21 days (according to the hygromycin B LD_50_, shown in Figure 1). The stable clones were isolated and screened by measuring their luciferase activity. As mentioned in Figure 2, luciferase activity was increased in transfected cells in normal condition without addition of any toxic chemical. Following treatment with different doses of toxins which disrupt cellular energy production (inhibit cellular respiration and ATP generation within the cell), luciferase activity decreases since ATP is an essential substrate for luciferase enzyme. No changes in the luciferase activity of Huh7-CMV-luc cells were measured over eight passages and after multiple rounds of storage in liquid nitrogen and re-culture. 

*Reduction of luciferase activity induced by chemical toxins in the biosensor*

When it was proved that the recombinant cells have high luciferase activity in normal condition without any induction, the dose dependent effects of chemical toxins on this cell line was investigated in order to evaluate their sensitivity to ATP decrement as a consequence of chemical toxicants. In this regard, the cells were seeded at a density of 2 × 10^5^ per well in 24-well microtiter plates, and incubated until the cells reached 70–80 % confluence. Then, they were treated with increasing concentrations of some common chemical food toxins such as nitrite, lead, xylene, and acrylamide. After 24 h, luciferase activity was measured according to the protocol. Data revealed that this toxin affects luciferase activity in a dose dependent manner, with maximal sensitivity a roximately about 0.2 μM to toxin concentrations (Figure 3 A-D).

*Shortest induction validation*

Decrement in luciferase activity by lead, nitrite, and xylene was also time dependent; it decreased 3-fold after 4 or 6 h, 4-fold after 12 h and 5-fold after 24 h of treatment with 0.1 to 10 μmol/L lead, nitrite and xylene. The luciferase activity decreased significantly after 4 h as the shortest induction time (*p* < 0.001). The time course of luciferase decrement by 0.1 to 10 μM of toxins in recombinant cell lines is shown in Figure 4.

## Discussion

These days food safety becomes a very important issue for human health. Environmental contamination or failures during food handling, processing, packing, and distribution can lead to biological, chemical, and/or physical threats. This increasing number of potentially harmful pollutants in the environment calls for accurate and sensitive detection of food contaminants. 

Researchers in diverse fields have developed numerous biosensors for a lications in agri-food industry. Conventionally, methods such as liquid and gas chromatography coupled with mass spectrometry and spectrophotometry are commonly used for monitoring toxicity. But these methods require expensive instruments, complex pretreatment steps, specialized personnel, and large quantity of organic solvents. Such methods cannot be used to perform in situ assays. 

Recent years have seen increasing interest in the a lication of simple, rapid, inexpensive and disposable chemical sensors for use in the fields of clinical, environmental or industrial analysis. In this context, biosensors a ear as a suitable alternative or as a complementary analytical tool. Biosensors and chemical sensors represent analytical devices that utilize the sensitivity and selectivity of a biomaterial, chemical compound or a combination of both attached onto the surface of a physical transducer for sensing purposes (6). Though whole cell-based biosensors are not as sensitive to environmental changes as molecular-based ones, these biosensors can be modified by simple genetic engineering methods so that they can then be used to detect a series of complex responses within a living cell. These biosensors can further provide information that molecule-based biosensors are not capable of, such as information related to the pharmacology, cell physiology, and toxicology of a sample. 

Light production in biological systems is called bioluminescence. The oxidation of firefly D-luciferin uses the energy from ATP conversion to AMP and i to form oxyluciferin, carbon dioxide, and light (7). Adenosine triphosphate (ATP), the basic energy source of the living cells, exists in all cells and determines the energy level of the cell. Therefore, the amount of ATP measured represents the number of living cells and describes the "well- being" of the cultured cells. 

In 1984, Kangas L *et al*. used bioluminescence to evaluate the drug effects by measuring the levels of ATP, which was significantly correlated with cell number and viability (8). After finding the bioluminescence as an efficient tool for assessing general toxicity, many studies used this a roach to generate whole cell biosensors in various fields. In 2000, Gil *et al*. generated a whole-cell bacterial biosensor for the detection of gas toxicity using a recombinant bioluminescent *Escherichia coli *(9), and in 2001, Farré *et al*. used a bacterial biosensor for assessment of organic pollutions in wastewaters (10), which are examples of the first generation of whole cell biosensors based on bacterial cells. Soon after, next generation of bioluminescence whole cell biosensors using eukaryotic cells such as yeast and fungi have emerged. For instance, Hollis *et al*. genetically modified *Saccharomyces cerevisiae* to express firefly luciferase, generating a bioluminescent yeast strain, and presence of any toxic chemical interfering with the cell′s metabolism resulted in a quantitative decrease in bioluminescence (11). 

However, eukaryotic cells such as *S. Cerevisiae* are more similar to human cells compared to the bacterial cells, and can evaluate a better estimation of toxicity of different components endangering humans, they still doesn’t represent the accurate risk of different toxins to human cells. Nowadays, researchers use differenet cell lines to generate whole cell biosensors as they are much more similar to human normal cells. In 2008, Banerjee *et al* . used B-cell hybridoma, Ped-2E9, encapsulated in type I collagen matrix to generate a biosensor for rapid detection of pathogens and toxins ( 12 ). In 2015, Motahari *et al* . generated a luminescence-based biosensor for detecting antioxidant and oxidant activities of samples using Huh7 cell line ( 13 ). In that study, huh7 cell line was stably transfected with the recombinant PGL4.26 plasmid containing *luc* gene under the transcriptional control of the ARE core sequence of the human NQO1 promoter in order to screen oxidative stress inducers. After treatments of huh7-ARE-luc cell line with oxidant agents, luciferase activity was increased, using "lights on" strategy, whereas here we use "lights off" strategy for detecting chemical toxins, which means that huh7-CMV-luc biosensor in this article has high luciferase activities in normal conditions and any ATP decrement results in reducing the luciferase activity.

In the following article, ATP produced by cellular biosensor is measured to detect microbial contamination in water and food. The hCMV enhancer, which seems to have little cell type or species preference, is several folds more active than the SV40 enhancer ( 14 ). It is one of the strongest enhancers analyzed so far, a property that makes it a useful component of eukaryotic expression vectors. The promoter-regulatory region of the major immediate early gene of human cytomegalovirus (hCMV) was sequenced in 1985. Boshart *et al.* ( 15 ) described the enhancer sequence essential for constitutive expression of downstream genes. According to this data, PGL4.26 plasmid, a vector containing the *luc* gene downstream of a minimal promoter was used. Then enhancer sequence of human cytomegalovirus (hCMV), which is a strong constitutive promoter cloned up the stream of *luc* gene in order to increase luciferase activity. Therefore, the cells containing this vector have high luciferase activity in normal conditions without any induction following to overexpression of *luc* gene. The pGL4.26-CMV-luc plasmid containing the Hygromycin B selectable marker was stably transfected into Huh7 cell line. After 21 day treatment with LD_50_ dose of hygromycin, and several passages of cells, a stable cell line was produced. Now this cell line will be able to detect the slightest changes in ATP level, due to the effect of different toxins on the cell which disrupt cellular respiration and ATP production processes. In order to investigate the sensitivity of the cell line, the cells were incubated with increasing concentrations of chemical food toxins affecting ATP production. These toxins affect luciferase activity in a dose dependent manner, with maximal sensitivity a roximately about 0.2 μM to toxin concentrations.

The huh7-CMV-luc stable cell line has the potential to be a unique biosensor because it is capable of detecting a wide range of toxic chemicals which are commonly poisoning food and cause damages. Hepatocellular carcinoma cell line share several characteristics of normal hepatocyte cells and offer stable phenotype and reproducibility, which make it useful for *in-vitro* drug safety analysis ( 16 ). Human hepatoma Huh-7 cell line is a proper model system to study liver metabolism and toxicity of xenobiotics; and the detection of antitoxic agents ( 17 ). The significant characteristic of Huh7 cells that makes them attractive in toxicity studies is that they express drug metabolizing enzymes. These cells display the functional activities of various carbohydrate metabolizing enzymes and represent an alternative model system for drug efficacy or toxicity studies related to the liver-specific genes induction ( 18 ). *In-vitro* liver cell lines are increasingly used for the toxicological studies due to the central role of the liver in chemical transforming and clearing.

In this article, the toxicity of several commonly used food toxicants was measured through luciferase assay. Due to the insertion of a strong enhancer sequence upstream of *luc* gene and overexpression of *luc* gene in normal condition, the whole cell biosensor (huh7-CMV-luc cell line) demonstrated high luciferase activity without any induction. After exposure of cell culture to different doses of contaminants, luciferase activity was measured to detect whether the contaminant is toxic and in which concentration may endanger the cell line. In each assay, the results were compared to the control culture with no chemical added to their culture medium. This process was done for about up to 10 chemicals and toxins in 0.1-10 μM range and in most of the cases, luciferase activity showed a roximately 5 fold decrease in 10 μM concentration. The responsiveness of this cell line to these substrates is in 0.1-0.2 μM range; which would imply high sensitivity. The levels of concern for any harmful component in various items (air, soil, food, blood, etc.) are set by the Food and Drug Administration (FDA) based on calculations of the amount that a person can consume without ill affect ( 19 ). For example, the FDA used a blood lead level (BLL) of 10 μg/dL (a roximately about 0.5 μmol/L) as the threshold for adverse effects in children and in pregnant or lactating women and BLL of 30 μg/dL (a roximately about 1.5 μmol/L) as a threshold level for adults, including non-pregnant women of childbearing age ( 20 ), both of which are easily detectable with this biosensor.

It is demonstrated that luciferase activity in Huh7-1x-CMV-luc cell line is dose dependent, in the presence of 3 μM of chemicals, cell line shows a ~ 2 fold decrease in luciferase activity and by increasing the concentration it reaches to ~ 3 fold decrease ( Figure 3, A-D ).

Since the biosensor measurements need to be verified and validated, there are several validation procedures accepted for quantitative methods, in the case of qualitative methods. Thevenot *et al* . recommended several analytical parameters to characterize biosensor performance, such as sensitivity, limit of detection (LOD), and selectivity and reliability ( 21 ). LOD (limit of detection), is the lowest quantity of a substance that can be distinguished from the absence of that substance (a blank value) with a stated confidence. LOD amounts for this method of detection were 0.5-1 µg/L for Lead, nitrite and acrylamide and 2 µg/L for xylene.

Data also revealed that the decrement in luciferase activity is also time-dependent. As illustrated in Figure 4 , the cell cultures were incubated for different time courses (4 h, 6 h, 12 h, and 24 h) to examine the shortest period induction. It was showed that Huh7-CMV-luc could stimulate a small, but a arent induction of luciferase activity as early as 4 h after treatment as a rapid detection.

## Conclusion

The need for disposable systems or tools for environmental a lications, in particular for toxicity monitoring, has encouraged a growing interest in the development of new technologies and more suitable methodologies. The use of biosensors technology for food safety will facilitate complying with international quality and safety standards, allowing for the efficient, safe, and reliable detection and quantification of inorganic contaminants that threaten consumer health. However, the detection of small concentrations of chemical and biological polluting substances in products for human and animal consumption is still needed (22). Bioluminescence is a fast (the results are seen directly within a few seconds), technically simple, sensitive, and flexible method (any cell line and drug may be studied by many different test designs). 

The most significant advantage of Whole cell biosensors is to provide information about the effect of a stimulus on a living system and detect the functional aspects of a host–hazard interaction in order to render an accurate estimation of the risks (23).

In many cases, functional rather than analytical information is ultimately desired. These biosensors provide the opportunity to elicit such information, for a lications such as pharmacology, cell biology, toxicology, and environmental measurements. 

The Huh7-CMV-luc cell line as a whole cell biosensor has selectivity, specificity, and rapid response compared to the previous biosensors for monitoring the cell toxicity. According to literature, it can be considered as a reproducible and reliable tool for *in-vitro* screening of cell responses to different concentrations of toxins and other compounds which disrupts the ATP generation mechanisms due to cellular damage. 
